# Hyperspectral Image Classification for Land Cover Based on an Improved Interval Type-II Fuzzy C-Means Approach

**DOI:** 10.3390/s18020363

**Published:** 2018-01-26

**Authors:** Hongyuan Huo, Jifa Guo, Zhao-Liang Li

**Affiliations:** 1 Key Laboratory of Agricultural Remote Sensing, Ministry of Agriculture/Institute of Agricultural Resources and Regional Planning, Chinese Academy of Agricultural Sciences, Beijing 100081, China; huohongyuan@caas.cn; 2 College of Geography and Environment, Tianjin Normal University, Tianjin 300387, China; 3 ICube, CNRS, Université de Strasbourg, 300 Boulevard Sébastien Brant, CS10413, 67412 Illkirch, France

**Keywords:** hyperspectral remote sensing, land cover, interval type-II fuzzy set, classification

## Abstract

Few studies have examined hyperspectral remote-sensing image classification with type-II fuzzy sets. This paper addresses image classification based on a hyperspectral remote-sensing technique using an improved interval type-II fuzzy c-means (IT2FCM*) approach. In this study, in contrast to other traditional fuzzy c-means-based approaches, the IT2FCM* algorithm considers the ranking of interval numbers and the spectral uncertainty. The classification results based on a hyperspectral dataset using the FCM, IT2FCM, and the proposed improved IT2FCM* algorithms show that the IT2FCM* method plays the best performance according to the clustering accuracy. In this paper, in order to validate and demonstrate the separability of the IT2FCM*, four type-I fuzzy validity indexes are employed, and a comparative analysis of these fuzzy validity indexes also applied in FCM and IT2FCM methods are made. These four indexes are also applied into different spatial and spectral resolution datasets to analyze the effects of spectral and spatial scaling factors on the separability of FCM, IT2FCM, and IT2FCM* methods. The results of these validity indexes from the hyperspectral datasets show that the improved IT2FCM* algorithm have the best values among these three algorithms in general. The results demonstrate that the IT2FCM* exhibits good performance in hyperspectral remote-sensing image classification because of its ability to handle hyperspectral uncertainty.

## 1. Introduction

The Earth’s land surface is a dynamic canvas on which human beings and natural systems are always interacting [1]. Land-use–land-cover (LULC) classification and its dynamics, which partially result from land-surface processes, have considerable effects on biotic diversity, soil degradation, terrestrial ecosystems, and the ability of biological systems to support human needs. These changes also have consequences for the radiation budget, resulting in profound effects on regional and global climates [2,3,4]. Thus, land-cover classification and its dynamics is an important field in environmental-change research at different scales. The efficient assessment and monitoring of land-cover changes are indispensable to advance our understanding of the mechanisms of change and model the effects of these changes on the environment and associated ecosystems at different scales [5,6,7,8,9,10].

Remote-sensing techniques represent some of the most effective tools to obtain information on LULC classification and dynamics (i.e., temporal-spatial changes and the transformation of landscapes) [7,11,12,13]. Many methods can detect land-cover changes based on optical and radar imagery with different spatial and spectral resolutions [14,15,16,17,18,19,20,21,22,23,24]. Existing techniques for accomplishing land-cover classification can be broadly grouped into three general types: (1) Supervised classification algorithms, such as the maximum likelihood, minimum distance, spectral angle mapping, and support vector machine methods, employ labeled training data or spectral measurements and ground-cover classes of interest; (2) Unsupervised classification methods, such as iterative self-organizing data analysis (ISODATA) techniques and k-means, are used to classify land-cover types without prior knowledge of the ground-cover classes of interest; (3) Combinations of supervised and unsupervised classification algorithms account for the remaining methods. These methods include an important assumption, namely, a pixel can only be classified into one category and the relationship between a pixel and type can only be a one-to-one relationship.

In some Boolean classification methods, e.g., the artificial neural network (ANN) method, an output node’s number corresponds to the number of pattern classes during the training course, and the output node that corresponds to the class of the training pattern vector is set to “1”, whereas all other output nodes are set to “0” [25]. In many hazardous situations, however, classes are often fuzzy or ill defined. Thus, most traditional classifiers often fail to provide an adequate representation of the relationship between a pattern vector and its ‘belongingness’ to a particular class [26]. Considering this aspect, an image pixel that corresponds to a ground entity does not represent only one category. Instead, this pixel corresponds to a mixture of two or more categories because of the resolutions of remote-sensing images and other factors. For example, a Thematic Mapper (TM) cell with dimensions of 30 m × 30 m that covers a portion of a residential area may include houses, meadows, and roads. If this cell is assigned a single type (house, grass, or road), the classification will contain significant errors. Classification algorithms that are based on fuzzy sets have been demonstrated to be more appropriate for land-cover change dynamics than most traditional Boolean classification algorithms [27,28,29].

A large of amount of high spatial resolution and hyperspectral remotely sensed data is becoming available due to the fast development of satellite and sensor technology, the above-mentioned supervised and unsupervised classification methods could swiftly obtain the clustering information from the remote-sensing data, thus, these algorithms play an important role in remote-sensing application. Recently, concerning the classification based on high spatial resolution and hyperspectral remote-sensing data, many machine-learning algorithms such as neural networks (NN), support vector machines (SVMs), and decision trees have been used to the process of classifying remotely sensed images [30,31,32]. However, most of the existing research work follows the traditional paradigm of pattern recognition. These algorithms used to image clustering consist of two steps: first, based on the raw data input, the complex handcrafted features are extracted, and second, the obtained features are used to learn classifiers. However, it is rarely known which features are important for the classification process due to the high diversity of depicted materials. Furthermore, for bigger datasets and many quite large remotely sensed images with very high spectral and spatial resolution, some deep learning methods or frameworks seems to more effectively fit and address the classification problems. The recent techniques based on deep learning have shown promising results for the classification of hyperspectral data like the convolutional neural network (CNN) and automatic encoder (AE) methods [33,34,35,36].

During image segmentation with fuzzy classification, a record of the degree to which any considered pixel belongs to a certain cluster is retained [37]. Traditional clustering algorithms, such as fuzzy c-means (FCM), kernel FCM, and k-means, are all type-I FCM classification methods. Most of these methods quantify the degree of similarity between the data points and the corresponding membership degree based on the Euclidean distance [29,38,39]. The FCM spatial information from FCM methods has been used to enhance algorithms to segment remote-sensing imagery in the presence of noise [40,41]. A novel semi-supervised fuzzy c-means (RSFCM) classification method was proposed to detect an increased proportion of changes and suppress noise through the synergistic exploitation of pseudo labels from difference images and spatial information [39]. An adaptive spatial-information-based fuzzy clustering method to segment an image that addresses sensitivity to noisy information and a lack of spatial information has proven to be helpful in improving the robustness of traditional FCM methods [42].

However, conventional type-I FCM classification methods, including the FCM, kernel FCM, and k-means approach, often display suboptimal performance when applied to data that exhibit complex geometry because they fail to handle and quantify uncertainty when determining their membership functions [43]. In contrast, the concept of a type-2 fuzzy set (TIIFS) was first introduced by [43] as an extension of the concept of an ordinary fuzzy set (henceforth called a type-1 fuzzy set (TIFS)). The membership degree of a TIFS is crisp, whereas a TIIFS is a “fuzzy-fuzzy set” because of its fuzzy membership degrees. Therefore, TIIFSs are particularly useful when determining an exact membership function for a fuzzy set is difficult; hence, TIIFSs have unique advantages in characterizing the uncertainty in hyperspectral image data from the sensors and other environmental factors, including the weather conditions. These sets are used for image classification via the interval type-II fuzzy c-means (IT2FCM) method [44,45,46,47]. 

At present, very few studies in the literature that focus on land-cover classification have employed FCM based on TIIFSs, especially with hyperspectral images. A fuzzy number refers to a connected set of possible values and is a generalization of a regular real number that does not refer to a single value. This behavior is a common natural phenomenon; in particular, the spectra of geographical features on the surface display similar behavior. The spectrum of one geographic feature is considered to be a connected set of possible and similar spectral curves, that is, a spectrum with a certain width, similar to a band. Existing FCM methods based on TIIFSs, e.g., interval type-II fuzzy c-means (IT2FCM), fail to consider the width of such bands, only using the ranking of the average values of the upper and lower membership degree to determine if the pixel under consideration belongs to a specific class, and these methods never consider the possibility-based interval-number ranking. 

Hence, according to above analysis, comparing with the FCM and IT2FCM, this paper will propose an improved interval type-II fuzzy c-means called IT2FCM* which improves on IT2FCM by incorporating interval number ranking methods, interval number distances, and water index to address the uncertainties for hyperspectral remote-sensing imagery clustering. This is the main objective of this study. In order to validate the separability of IT2FCM* algorithm comparing to two other fuzzy methods, FCM and IT2FCM, four clustering validity indexes are used: the partition coefficient index (PC), the Fukuyama and Sugeno index (FS), the Xie and Beni index (XB), and the partition entropy (PE). These validity indexes for the FCM, IT2FCM, and IT2FCM* algorithms are calculated based on different spectral and spatial resolution remotely sensed datasets. As a second objective of this paper, a comparative analysis of variation of their values is made to show and judge the performance of these three clustering fuzzy algorithms.

## 2. Methodology 

**Figure 1:**
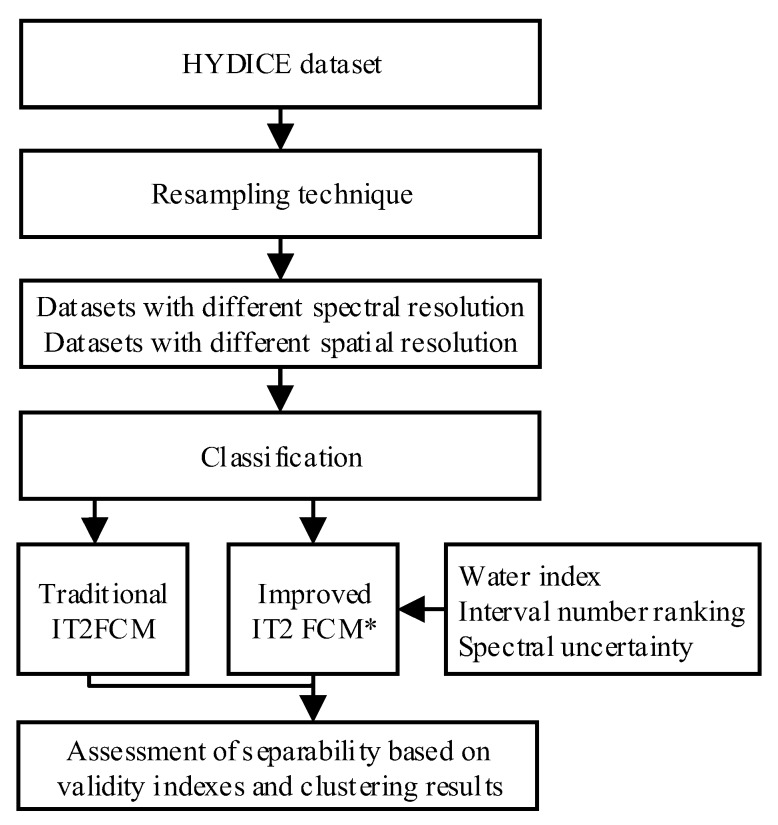
Flowchart that shows land-cover classification based on hyperspectral remote-sensing datasets with different spectral and spatial resolutions. HYDICE, Hyperspectral Digital Imagery Collection Experiment; IT2FCM, interval type-II fuzzy c-means.

In this section, the IT2FCM algorithms for land-cover clustering are briefly described. The spectral uncertainty and ranking of interval numbers were considered in the improved IT2FCM* algorithm as improvements to the IT2FCM algorithm. The resampled remotely sensed datasets with different spectral and spatial resolutions were then used for land-cover classification by using the IT2FCM* algorithm, and then four different indexes were used to evaluate the separability of the improved IT2FCM* algorithm. A detailed flowchart that describes this study is shown in Figure 1.

### 2.1. Water Index

Given the high spatial and spectral resolution of the remote-sensing datasets in this study, these data contained many shadows that were cast by features such as tall buildings and trees. However, the spectral signature of water is often and easily confused with the spectral features of other dark objects (e.g., shadows), which is a common phenomenon in urban-classification research. Therefore, we separated water from shadows within the study area before the remote-sensing data with different spectral and spatial resolutions were classified with the IT2FCM* algorithm. The modified normalized difference water index (MNDWI) was employed and incorporated into the IT2FCM* method to mask these water areas and identify them from shadow areas. For details on the calculation of the MNDWI, see [47,48]. As mentioned above, the spectral signature of water is often and easily confused with the spectral features of other dark objects (e.g., shadows), which is a common phenomenon in urban-classification research. Thus, the modified normalized difference water index (MNDWI) [47,48] was employed and incorporated into the improved IT2FCM* algorithm to automatically mask water areas. The MNDWI is expressed as follows:

(1) $$MNDWI=\frac{\rho_{Green}-\rho_{MIR}}{\rho_{Green}-\rho_{MIR}}$$

where $\rho_{Green}$ is a spectral band that reflects green light (around 560 nm, corresponding to the band 37 of Hyperspectral Digital Imagery Collection Experiment (HYDICE) dataset), and $\rho_{MIR}$ is a spectral band that represents reflected middle-infrared radiation (around 1650 nm, corresponding to the band 120 of HYDICE dataset). When Equation (1) is used to process hyperspectral remote-sensing images, the values of water features are positive, while the values of soil and terrestrial features are zero or negative because of their typically higher reflectance values in the MIR spectral band than the values of green light. This relationship is very helpful to discriminate water areas from other low-reflectance targets, such as shadows. 

### 2.2. Description of the IT2FCM Clustering Algorithm

**Figure 2:**
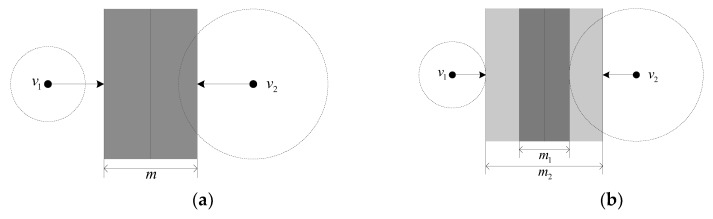
Descriptions of the FCM and IT2FCM. (**a**) Standard FCM method with a single fuzzifier *m*; (**b**) Classical IT2FCM approach with two fuzzifiers (*m*1 and *m*2).

The fuzzifier *m* and the number of classes *C* are the two parameters that can be set by users in most FCM algorithms [49]. The fuzzy membership grades, which measure a sample that belongs to a specific clustering class, may differ depending on the value of the fuzzifier *m* for *C*, a certain classification number [29,50]. The cluster centers are expressed by real-number vectors, and the distance between a sample and the cluster centers is used to determine the membership grade of a sample that belongs to one class, as shown in Figure 2 The vertical line in Figure 2a can be considered a “decision” boundary, and samples are located to the left or right side of the boundary. The maximum fuzzy boundary widens when the value of the fuzzifier *m* increases. Therefore, once the classification number C is determined, the fuzzy membership grade of a sample that belongs to a specific class is likely to be different if the fuzzifier *m* takes different values [29,50]. The same object may exhibit a different spectrum because of influences from multiple factors. Therefore, the same spectrum in a satellite image may correspond to a different classification in the natural or real world [51]. In other words, the spectrum centers of geographical features usually contain uncertainty. However, classical FCM clustering algorithms cannot handle this type of uncertainty.

Hwang and Rhee (2007) proposed the IT2FCM algorithm based on TIIFSs. This algorithm is used to address the uncertainty in choosing the fuzzier *m* (see Figure 2b). The lower and upper membership grades and functions are used to handle the fuzzy membership value’s uncertainty [51]. Two fuzzifiers *m*1 and *m*2, which are set by the users, are used to construct the upper and lower membership functions in IT2FCM. The two objective functions of the IT2FCM algorithm are then expressed through the following equations:

(2) $$\{\begin{array}{c} O_{m1}\left(U,v\right)=\sum_{k=1}^{M}\sum_{i=1}^{T}{\left(u_{ik}\right)}^{m1}d_{ik}^{2}\\ O_{m2}\left(U,v\right)=\sum_{k=1}^{M}\sum_{i=1}^{T}{\left(u_{ik}\right)}^{m2}d_{ik}^{2} \end{array}$$

where $d_{ik}$ equals the value of $∥x_{k}-v_{i}∥$, which is the distance metric between the cluster centroid $v_{i}$ and the sample $x_{k}$; *M* is the number of samples; and *T* is the number of classes. ${\bar{u}}_{ik}$ and ${\underline{u}}_{ik}$ are the upper and lower membership grades, respectively, and are expressed as follows:

(3) $$\left\{ \begin{array}{c} {\bar{u}}_{ik}=\{\begin{array}{c} \frac{1}{\sum_{j=1}^{T}{\left(d_{ik}/d_{jk}\right)}^{2/\left(m1-1\right)}}\;\mathrm{when}\;\frac{1}{\sum_{j=1}^{T}\left(d_{ik}/d_{jk}\right)}<\frac{1}{T}\\ \frac{1}{\sum_{j=1}^{T}{\left(d_{ik}/d_{jk}\right)}^{2/\left(m2-1\right)}}\;\mathrm{when}\;\frac{1}{\sum_{j=1}^{T}\left(d_{ik}/d_{jk}\right)}\geq \frac{1}{T} \end{array}\\ {\underline{u}}_{ik}\{\begin{array}{c} \frac{1}{\sum_{j=1}^{T}{\left(d_{ik}/d_{jk}\right)}^{2/\left(m1-1\right)}}\;\mathrm{when}\;\frac{1}{\sum_{j=1}^{T}(d_{ik}/d_{jk})}\geq \frac{1}{T}\\ \frac{1}{\sum_{j=1}^{T}{\left(d_{ik}/d_{jk}\right)}^{2/\left(m2-1\right)}}\;\mathrm{when}\;\frac{1}{\sum_{j=1}^{T}(d_{ik}/d_{jk})}<\frac{1}{T} \end{array} \end{array}\right.$$

(4) $$v_{i}=\sum_{k=1}^{M}{\left(u_{ik}\right)}^{m}x_{k}/\sum_{k=1}^{M}{\left(u_{ik}\right)}^{m}$$

where *k* = 1, 2, …, *M* and *i* = 1, 2, …, *T*. $v_{i}$ is the cluster centroid, which is represented by the interval between *v^L^* and *v^R^*, and is computed in the same manner as in most FCM methods. *v^L^* and *v^R^* are determined by the Karnik-Mendel (KM) algorithm from [52], and $v_{i}$ is obtained by type reduction:

(5) $$v_{i}=(v_{i}^{L}+v_{i}^{R})/2.$$

The membership grades $u_{ij}$ can then be calculated by the following equation:

(6) $$u_{ij}=(u_{ij}^{L}+u_{ij}^{R})/2$$

where *i* = 1, 2, …, *T* and *j* = 1, 2, …, *M*. $u_{ij}^{L}$ and $u_{ij}^{R}$ are determined by the following two equations:

(7) {uijL=∑l=1muijlN and uijl={u¯ij when xil uses u¯ij for vLu_ij otherwiseuijR=∑l=1muijlN and uijl={u¯ij when xil uses u¯ij for vRu_ij otherwise

where *N* is the number of the samples’ features. Then, the class to which a sample belongs depends on the rule that if *u_ik_* > *u_jk_* for *i* ≠ *j* and *j* = 1, 2, …, *T*, then the sample *x_k_* is assigned to the cluster *i*.

According to the above analysis, the cluster centers are often expressed as real vectors in these existing FCM methods, so the errors of these cluster centers cannot be effectively handled. Therefore, the interval centroids of all the clusters should be type reduced to single values first. Moreover, some information will be lost when the type-II fuzzy membership functions are defuzzified into type-I fuzzy membership functions during each iteration [53]. The IT2FCM method and almost all its derived algorithms, such as IT2FCM clustering with spatial information (IIT2-FCM) [36], kernel interval-valued FCM (KIFCM) [42], interval type-II fuzzy possibility c-means (IFPCM) [54], interval-valued possibility fuzzy c-means (IPFCM) [55], and the general T2FCM (GT2 FCM) [56], often have the same faults. However, the characteristic spectra of a geographical feature should be a connected set of spectral curves, similar to a band with a certain width, and not only one spectral curve [57]. Errors from the data acquisition process (including from the sensors) and the processing, conversion, and transmission of the data may result in substantial uncertainties in the remote-sensing data [58].

### 2.3. IT2FCM* Algorithm

As is mentioned above, two fuzzifiers *m*1 and *m*2 are used to construct the maximum and minimum membership functions; however, there are still some faults in the IT2FCM algorithm (which are discussed in above section). Therefore, the improved IT2FCM based on interval number distance and ranking is proposed in this paper. Here, distance between the interval numbers can be defined by, e.g., the Euclidean distance, which is commonly used in other classification methods. However, this distance definition only considers the endpoint of the interval numbers. In this paper, the interval-number distance, which was proposed by [59], is adopted here in the IT2FCM* approach because it produces the best results among the existing interval-number distance methods:

(8) $$\tilde{D}\left({\bar{a}}_{1},{\bar{a}}_{2}\right)=\left(\frac{a_{1}^{-}+a_{1}^{+}}{2}-\frac{a_{2}^{-}+a_{2}^{+}}{2}\right)+\frac{1}{3}\left[{\left(\frac{a_{1}^{+}-a_{1}^{-}}{2}\right)}^{2}+{\left(\frac{a_{2}^{+}-a_{2}^{-}}{2}\right)}^{2}\right]-\frac{1}{6}{\left[{\left({\bar{a}}_{1}\cap {\bar{a}}_{2}\right)}^{+}-{\left({\bar{a}}_{1}\cap {\bar{a}}_{2}\right)}^{-}\right]}^{2}$$

where ${\bar{a}}_{1}=\left[a_{1}^{-},a_{1}^{+}\right]$ and ${\bar{a}}_{2}=\left[a_{2}^{-},a_{2}^{+}\right]$; both are two-interval numbers.

In the IT2FCM* algorithm, all the cluster centroids are interval-number vectors. Hence, the Euclidean distance between an interval-cluster centroid and a sample can be measured by the following equation:

(9) $$\tilde{D}\left(x,\tilde{v}\right)={\left(\sum_{i=0}^{N}D^{2}\left(x_{i},{\tilde{v}}_{i}\right)\right)}^{\frac{1}{2}}={\left(\sum_{i=0}^{M}{\left(x_{i}-\frac{{\tilde{v}}_{i}^{-}+{\tilde{v}}_{i}^{+}}{2}+\frac{1}{3}{\left(\frac{{\tilde{v}}_{i}^{+}-{\tilde{v}}_{i}^{-}}{2}\right)}^{2}\right)}^{2}\right)}^{\frac{1}{2}}$$

where $\tilde{v}$ is an interval-number vector, *x* is a sample, *i* = 1, 2, …, *N*, and *N* represents the number of features. An interval-number vector ${\tilde{v}}_{i}$ in $\tilde{v}$ has a lower and upper bound ${\tilde{v}}_{i}^{-}$ and ${\tilde{v}}_{i}^{+}$, respectively. The upper and lower membership grades of each sample ${\bar{u}}_{ik}$ and ${\underline{u}}_{ik}$ can be calculated by the two following equations based on two different fuzzifiers *m*1 and *m*2 and the interval-number distance methods:

(10) $$\left\{ \begin{array}{c} {\bar{u}}_{ik}=\{\begin{array}{c} \frac{1}{\sum_{j=1}^{T}{\left({\tilde{d}}_{ik}/{\tilde{d}}_{jk}\right)}^{2/\left(m1-1\right)}}\;\mathrm{when}\;\frac{1}{\sum_{j=1}^{T}\left({\tilde{d}}_{ik}/{\tilde{d}}_{jk}\right)}<\frac{1}{T}\\ \frac{1}{\sum_{j=1}^{T}{\left({\tilde{d}}_{ik}/{\tilde{d}}_{jk}\right)}^{2/\left(m2-1\right)}}\;\mathrm{when}\;\frac{1}{\sum_{j=1}^{T}\left({\tilde{d}}_{ik}/{\tilde{d}}_{jk}\right)}\geq \frac{1}{T} \end{array}\\ {\underline{u}}_{ik}\{\begin{array}{c} \frac{1}{\sum_{j=1}^{T}{\left({\tilde{d}}_{ik}/{\tilde{d}}_{jk}\right)}^{2/\left(m1-1\right)}}\;\mathrm{when}\;\frac{1}{\sum_{j=1}^{T}({\tilde{d}}_{ik}/{\tilde{d}}_{jk})}\geq \frac{1}{T}\\ \frac{1}{\sum_{j=1}^{T}{\left({\tilde{d}}_{ik}/{\tilde{d}}_{jk}\right)}^{2/\left(m2-1\right)}}\;\mathrm{when}\;\frac{1}{\sum_{j=1}^{T}({\tilde{d}}_{ik}/{\tilde{d}}_{jk})}<\frac{1}{T} \end{array} \end{array}\right.$$

where *k* = 1, 2, …, *M* and *i* = 1, 2, …, *T*.

According to Equation (2), the two objective functions and the pre-determined condition can be expressed as the following equations:

(11) $$\{\begin{array}{c} Q_{m1}\left(U,v\right)=\sum_{k=1}^{M}\sum_{i=1}^{T}{\left(u_{ik}\right)}^{m1}d_{ik}^{2}\\ Q_{m2}\left(U,v\right)=\sum_{k=1}^{M}\sum_{i=1}^{T}{\left(u_{ik}\right)}^{m2}d_{ik}^{2} \end{array}$$

(12) $$Q_{m}^{c+1}\left(U,v\right)-Q_{m}^{c}\left(U,v\right)\leq \sigma .$$

The KM algorithm was adopted here to determine $v_{i}^{L}$ and $v_{i}^{R}$. The iteration is stopped if the equation $Q_{m}^{c+1}\left(U,v\right)-Q_{m}^{c}\left(U,v\right)$ is satisfied. The possibility-ranking method between interval numbers, which was first proposed by [60], was adopted in IT2FCM* algorithm.

The lower and upper membership grades of each sample that belong to each class are expressed by the interval-number vector $\tilde{u}$. An interval-number vector can be expressed as follows:

(13) $$\tilde{u}=\left\{{\tilde{u}}_{1k},{\tilde{u}}_{2k},\ldots ,{\tilde{u}}_{Tk}\right\}=\left\{\left[{\underline{u}}_{1k},{\bar{u}}_{1k}\right],\left[{\underline{u}}_{2k},{\bar{u}}_{2k}\right],\ldots ,\left[{\underline{u}}_{Tk},{\bar{u}}_{Tk}\right]\right\}.$$

Then, we can calculate the probability for any two intervals in the vector as follows:

(14) $$P({\tilde{u}}_{ik}\geq {\tilde{u}}_{jk})=\left\{ \begin{array}{c} 1\;\mathrm{where}\;{\underline{u}}_{jk}\leq {\bar{u}}_{jk}\leq {\underline{u}}_{ik}\leq {\bar{u}}_{ik}\\ 1-\frac{{\left({\bar{u}}_{jk}-{\underline{u}}_{ik}\right)}^{2}}{2L\left({\tilde{u}}_{ik}\right)L\left({\tilde{u}}_{jk}\right)}\;\mathrm{where}\;{\underline{u}}_{jk}\leq {\underline{u}}_{ik}\leq {\bar{u}}_{jk}\leq {\bar{u}}_{ik}\\ \frac{{\underline{u}}_{ik}+{\bar{u}}_{ik}-2{\underline{u}}_{jk}}{2L\left({\tilde{u}}_{jk}\right)}\;\mathrm{where}\;{\underline{u}}_{jk}\leq {\underline{u}}_{ik}\leq {\bar{u}}_{ik}<{\bar{u}}_{jk}\\ \frac{2{\bar{u}}_{ik}-({\underline{u}}_{jk+}{\bar{u}}_{jk})}{2L\left({\tilde{u}}_{ik}\right)}\;\mathrm{where}\;{\underline{u}}_{ik}\leq {\underline{u}}_{jk}\leq {\bar{u}}_{jk}\leq {\bar{u}}_{ik}\\ \frac{{\left({\bar{u}}_{ik}-{\underline{u}}_{jk}\right)}^{2}}{2L\left({\tilde{u}}_{ik}\right)L\left({\tilde{u}}_{jk}\right)}\;\mathrm{where}\;{\underline{u}}_{ik}<{\underline{u}}_{jk}\leq {\bar{u}}_{ik}<{\bar{u}}_{jk}\\ 0\;\mathrm{where}\;{\underline{u}}_{ik}<{\bar{u}}_{ik}<{\underline{u}}_{jk}<{\bar{u}}_{jk} \end{array}\right.$$

(15) $$\{\begin{array}{c} L\left({\tilde{u}}_{jk}\right)={\bar{u}}_{jk}-{\underline{u}}_{jk}\\ L\left({\tilde{u}}_{ik}\right)={\bar{u}}_{ik}-{\underline{u}}_{ik} \end{array}$$

where $L\left({\tilde{u}}_{ik}\right)$ and $L\left({\tilde{u}}_{jk}\right)$ are the widths of the interval numbers ${\tilde{u}}_{ik}$ and ${\tilde{u}}_{ik}$, respectively; for *i*, *j* = 1, 2, …, *T* and *k* = 1, 2, …, *M*.

**Figure 3:**
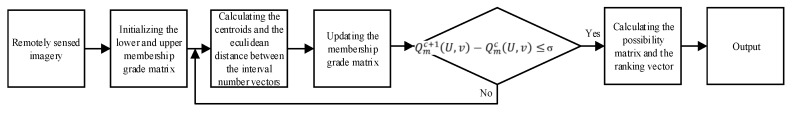
Diagram of the EnIT2FCM algorithm.

The possibility matrix can be obtained from the above equation, and its expression is as follows: P = (*pij, k*). Then, the ranking vector $w_{k}={\left(w_{1k},w_{2k},\ldots ,w_{Tk}\right)}^{Transpose}$ can be calculated by $w_{i}=\frac{1}{n\left(n-1\right)}\left(\sum_{j=1}^{n}p_{ij}+\frac{n}{2}-1\right)$ (*n*, interval numbers). Finally, the index of the maximum value in the ranking vector wk is the class index of the sample. A detailed flowchart of the improved IT2FCM* method is shown in Figure 3. Figure 3 clearly describes that the main steps of this algorithm includes essentially three steps. Firstly, just like other unsupervised algorithms, the number of image clustering types, the two fuzzifiers *m*1 and *m*2, and termination criterion value σ are given to initialize the minimum and maximum membership grade matrix on the basis of a random method, then all the centroids are calculated and membership grade matrix is update d [61]; Secondly, using the interval number ranking technique, classify each sample to a cluster according to the index of the maximum value in the ranking vector; Thirdly, report the clustering results, this step produces the best classification results based on the second step (here, although the outputs are crisp classification results, they are based on the index of the maximum value in the ranking vector and the optimal fuzzy membership value). The detailed information for these three steps is described by Table 1.

### 2.4. Validation of Clustering Results

Regarding the validation of the clustering results based on the improved IT2FCM* method and other two fuzzy methods, FCM and IT2FCM, considering the typical hyperspectral dataset used in this paper are provided together with corresponding ground truth dataset, thus, based on these ground truth data, the validation job is done, and the post-classification processing such as calculating the confusion matrix and accuracy of classification is finished. More details and specific information are described in experimental results.

### 2.5. Interval Type-1 Fuzzy Cluster Validity Index

In this paper, four cluster validity indexes were chosen to validate and demonstrate the separability of the IT2FCM* algorithm. The PC index, which was proposed by Bezdek [49], indicates the average relative amount of membership sharing between pairs of fuzzy subsets [62]. Thus, higher PC indexes correspond to better clustering results. The FS index, which was proposed by [63], was developed to measure the discrepancy between fuzzy separation and fuzzy compactness. The PE index, which was proposed by [49], is a scalar measurement of the amount of fuzziness in a set of classification results. The XB index, which was proposed by [64], is used to compare the average within-cluster fuzzy compactness to the minimum between-cluster separation [65]. Therefore, smaller PE, FS, and XB indexes indicate better classification performance.

## 3. Experimental Results

Considering the improved IT2FCM* algorithm is proposed for hyperspectral remotely sensed imagery clustering. It is necessary to use some famous and well-known hyperspectral datasets such as the Pavia University datasets, Washington HYDICE datasets, and so on. These hyperspectral datasets are all airborne remote-sensing imagery. To test the separability of the proposed IT2FCM* algorithm applying the satellite hyperspectral dataset, the EO-1 Hyperion satellite dataset is used in this study due to its free cost for downloading from the NASA official website. These datasets were used to test the accuracy of classification based on FCM, IT2FCM, and IT2FCM*. In this section, the membership values are firstly calculated from the IT2FCM* algorithm before classifying these images. Using the interval-number-ranking technique based on fuzzy membership values of different land cover types, the results with optimal membership fuzzy value are reported; the corresponding classification results from these three different remotely sensed datasets were done based on the FCM, IT2FCM, and IT2FCM* algorithms. 

### 3.1. Images of Membership Values of Different Classes

**Figure 4:**
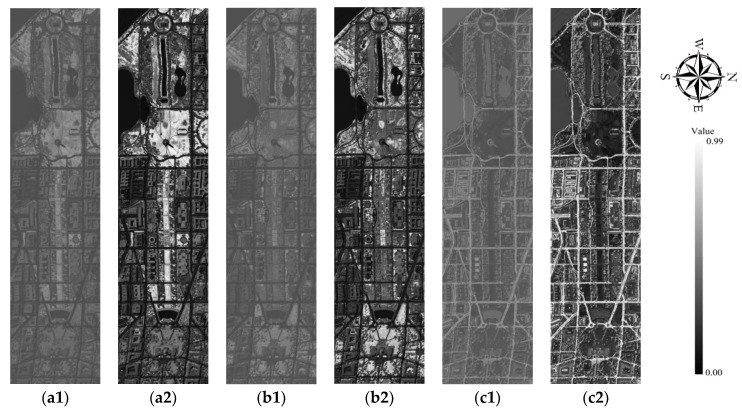

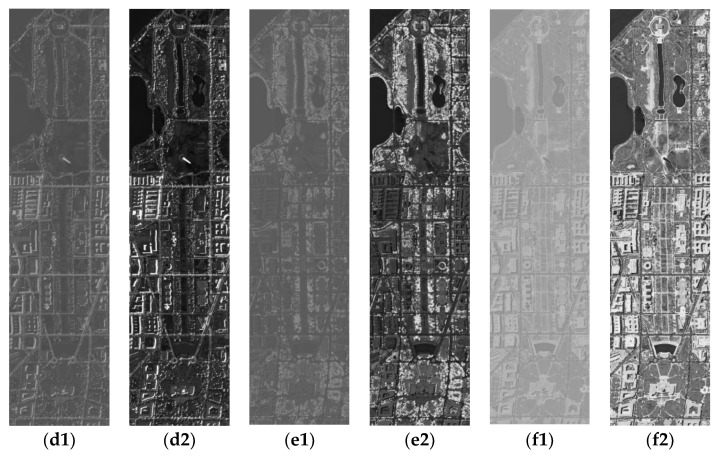
Images of membership values of different classes from IT2FCM* algorithm for a 191-band HYDICE dataset with a spatial resolution of 3 m. (**a1**–**f2**) are, respectively, the minimum and maximum membership values of sparse grass land, dense grass land, roads, shadows, trees, and bare soil and buildings.

In this part, many membership values maps are produced from above three remotely sensed datasets from the IT2FCM* algorithm; here, the 191-band hyperspectral HYDICE dataset with a spatial resolution of 3 m is taken as the example, and its membership values are calculated. The maximum and minimum membership values of different classes are shown in Figure 4. From the maximum membership value images shown in maps (see Figure 4(a2,b2,c2,d2,e2,f2)), it is obvious that almost all of the classes are well classified. Even these classes do not exhibit a minimum fuzzy membership (see Figure 4(a1,b,c1,d1,e1,f1)) close to 0. In fact, the maximum fuzzy membership values shown in Figure 4 of all classes are close to 1 in all maps. From this result, it is notably that all these land cover types were clearly differentiated. 

### 3.2. HYDICE Dataset and Classification Results

**Figure 5:**
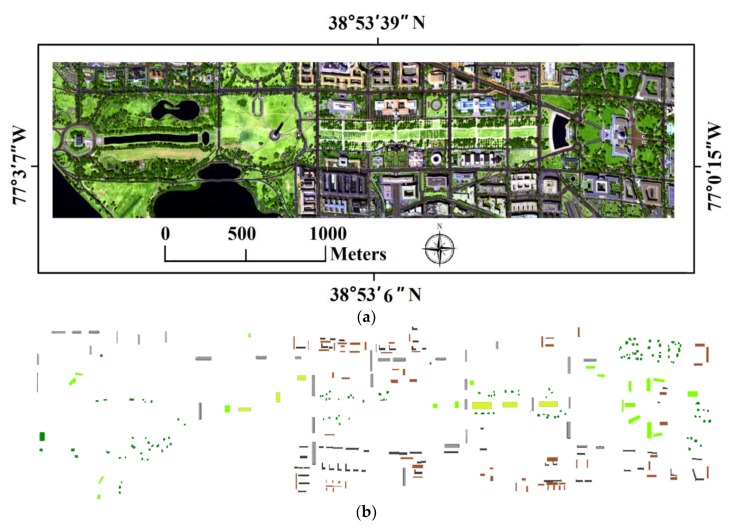

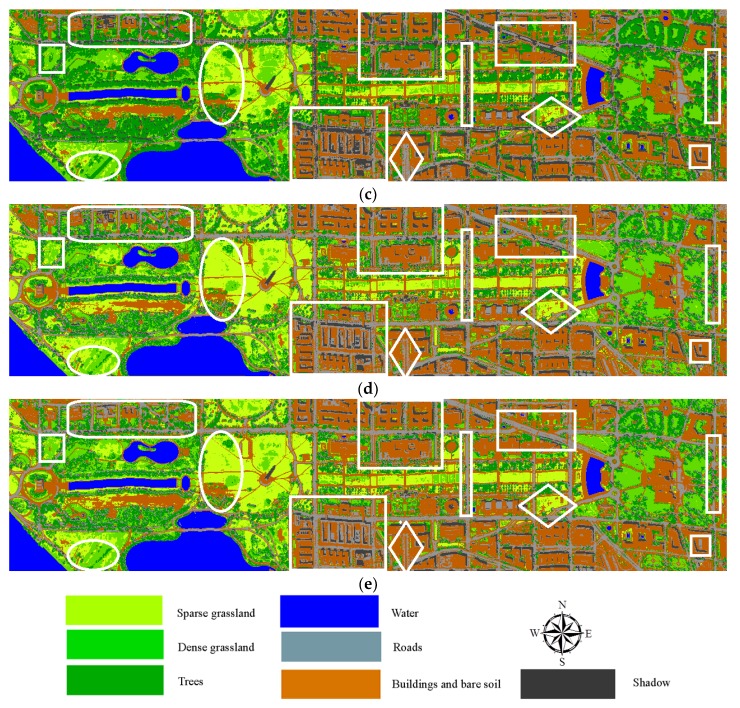
HYDICE dataset of study area, the samples, and classification results. (**a**) is a false-color composite image of study area that was constructed from bands 63, 52, and 36 (red, green, and blue, respectively). (**b**) shows the reference data for this study. (**c**) is the classification results from FCM, (**d**) is the results of classification from IT2FCM, and the (**e**) is the results of classification from the improved IT2FCM* method.

The Hyperspectral Digital Imagery Collection Experiment (HYDICE) hyperspectral dataset is a 191-band raw digital number hyperspectral image. The study area is located in the Washington D.C. Mall area in the U.S.A. This dataset was collected by the HYDICE sensor on 23 August 1995 (see Figure 5a). As a push broom aircraft sensor system, the HYDICE instrument operates within the spectral range from 400 to 2500 nm with 210 spectral bands. The spectral resolution of the HYDICE sensor is approximately 10 nm [66]. After several noisy bands were removed, the final image contained 191 spectral bands [40]. For more information, see [67]. The land cover within the study area was classified into six types by using the improved IT2FCM* algorithm. These types were sparse grassland, dense grassland, trees, bare soil and buildings, roads, and shadows (see Figure 5b). As is mentioned in above section, the water is masked, thus, during the post-classification processing, the water is incorporated into the results of classes, then the results of classes of the study area were organized into seven classes including sparse grassland, dense grassland, trees, water, bare soil and buildings, roads, and shadows.

To validate the results of land-cover classification based on the IT2FCM* algorithm, the ground truth dataset is collected, and the testing image and reference data are provided in Figure 5b. The confusion matrix was calculated based on regions of interest (ROIs). The accuracy of classification results (see Figure 5c–e) based on the FCM, IT2FCM, and IT2FCM* algorithms are calculated based on these matrixes (see Table 2). We also calculated the overall accuracy (OA) and kappa coefficient (KC) from the confusion matrix. The results showed that the overall accuracy of the results when using the image with 191 bands was 96.2% and the kappa coefficient was 0.95. Moreover, we conducted a comparative analysis of the classification results with higher-spatial-resolution aerial image data to further show the performance of the improved IT2FCM* algorithm.

Notably, comparing these three maps of classification results, it can be found that the classification map based on the improved IT2FCM* algorithm is the finest. Within the region in the white circle, regarding to the results of FCM and IT2FCM, some dense grassland are mistaken as the trees, while the results of IT2FCM* method is more fit the practical situation. Comparing these maps, within the regions in the white rectangle, it is notable that the shadows classified by the FCM and IT2FCM algorithm are more than those by the IT2FCM* algorithm, as some parts of dark buildings are mistaken as shadows, and typically, in some parts of road, the shadows are over estimated by the FCM and IT2FCM methods. Within the regions in the white diamond, results of FCM and IT2FCM algorithm, some parts of roads are mistaken as the buildings. Thus, in general, from the classification results, the IT2FCM* algorithm has the best performance with the hyperspectral HYDICE datasets.

### 3.3. Pavia University Dataset and the Classification Results

**Figure 6:**
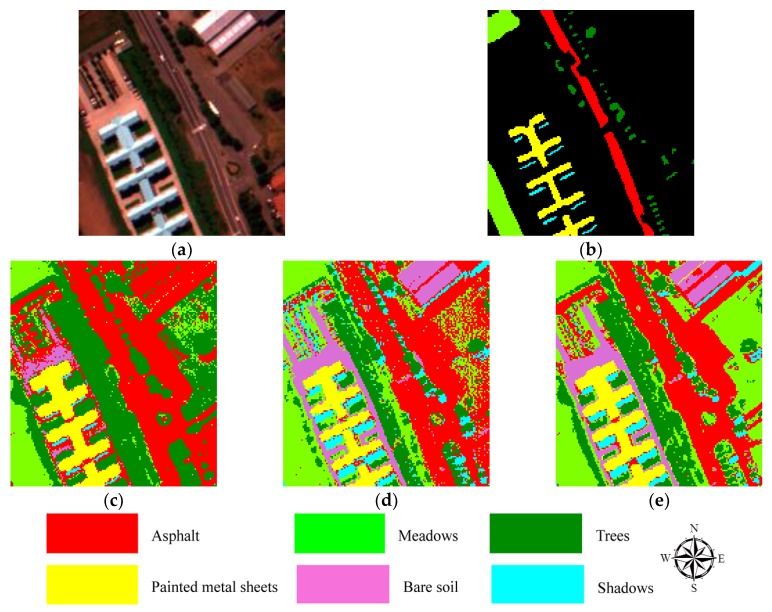
The study area and the classification results from three different fuzzy algorithms. (**a**) is the natural color composite image with band 60/32/10 for RGB of study area of Pavia University; (**b**) is the ground truth data used for estimating the accuracy of the classification results from these three different fuzzy algorithms; (**c**) is the classified results from FCM; (**d**) is the classified results from IT2FCM; and (**e**) is the results classified based on IT2FCM*.

The Pavia University dataset was captured by reflective optics spectrographic imaging system (ROSIS) airborne instrument on the city of Pavia (see Figure 6a). This instrument has 115 spectral channels with spectral region covering from 0.43 to 0.86 um, and the spatial resolution is 1.3 m per pixel. Due to the impacts of noise, 12 channels have removed, and the remaining 103 bands are further processed, and atmospheric correction was done [31,68]. This airborne dataset covers an area of the Engineering School of Pavia University, which consists of nine different classes, including asphalt, bitumen, metal sheet, gravel, bricks, soil, shadow, meadow, and trees. 

The Pavia University hyperspectral remote-sensing dataset is classified based on the three fuzzy clustering methods; the results are shown by Figure 6c–e. From Figure 6, comparing Figure 6d and 6e, the accuracy of FCM method is much lower than that of the other two fuzzy algorithms. To evaluate the results of classification, the ground truth ground dataset (see Figure 6b) was used, and the confusion matrix was calculated. Then, the accuracy was estimated based on the confusion matrixes (see Table 3). From Table 3, the accuracy value of IT2FCM* algorithm is the highest among these three methods, and almost all the values of accuracy of each class are notably higher than those of the other two fuzzy means. This result indicates the IT2FCM* algorithm has a good performance with the Pavia University hyperspectral remote-sensing data.

### 3.4. Hyperion Dataset and Classification Results

**Figure 7:**
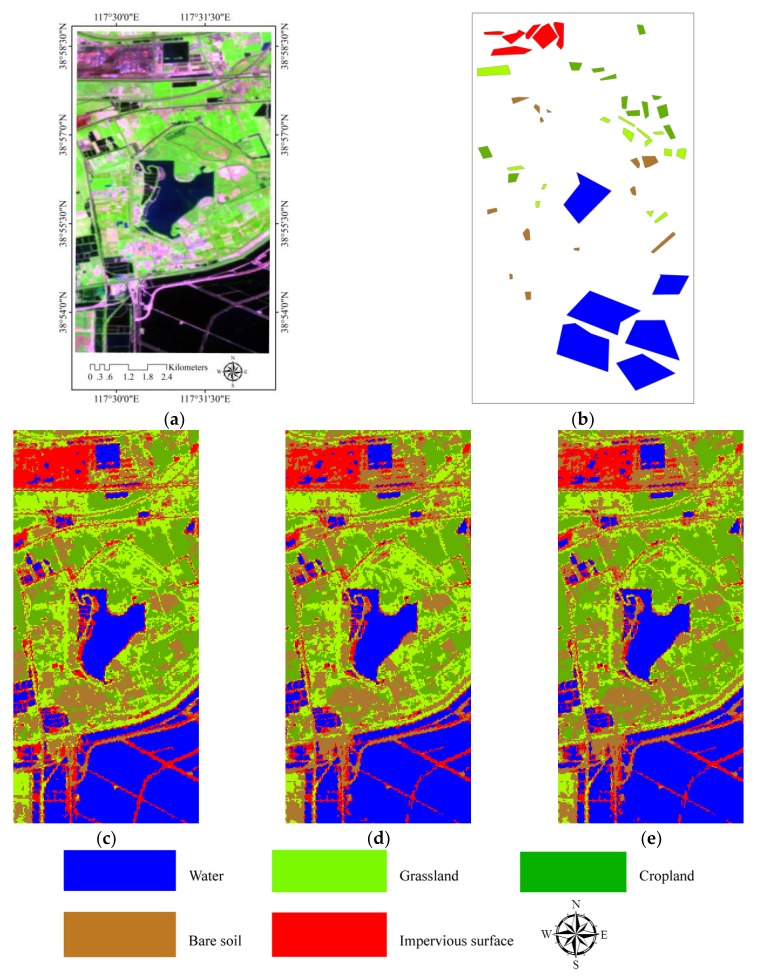
The study area and the classification results from three different fuzzy algorithms. (**a**) is the false color composite image with band 151/52/32 for RGB of study area from Hyperion dataset; (**b**) is the ground truth data used for estimating the accuracy of the classification results from these three different fuzzy algorithms; (**c**) is the classified results from FCM; (**d**) is the classified results from IT2FCM; and (**e**) is the results classified based on IT2FCM*.

This section introduces a satellite hyperspectral remote-sensing images, Hyperion image, acquired by Hyperion instrument, board on EO-1 satellite. Considering the free cost of the dataset and easy access to downloading, the Hyperion hyperspectral image of study area of Tianjin, north China, is used. The Hyperion instrument is a high-resolution hyperspectral imager capable of resolving 242 spectral bands ranging from 0.4 to 2.5 μm with a 30 m resolution. This instrument images a 7.5 km × 100 km surface area [69]. During the processing of the Hyperion image, after removal of the bad spectral bands, calibration, and the atmospheric and geometric correction, about 179 bands remained. The relating algorithms for calibration and atmospheric and geometric correction are provided in [70,71]. From the 179-band image, a further reduced set of ‘stable’ bands could be selected for further analysis. The basis for selection of these ‘stable’ Hyperion bands and the set of stable bands are provided by [72]. Figure 7a shows the study area. The main land cover of this study area includes water, grassland, cropland, bare soil, and impervious surface. To evaluate the accuracy of the classification results, the ground truth data was collected (see Figure 7b).

The landscape of the study area is classified into water, grassland, cropland, bare soil, and impervious surface based on Hyperion images from the FCM, IT2FCM, and IT2FCM* algorithms, which are shown in Figure 7c–e. To estimate the accuracy of the results from the above three fuzzy methods, the confusion matrix were calculated, the accuracy of each class, OA and KC, are also measured based on these confusion matrix (see Table 4). This table notably shows that the accuracy of IT2FCM* is higher than the other two fuzzy means and has a great improvement compared to the accuracy of the FCM method. 

## 4. Discussion

### 4.1. Consistency Testing Based on Validity Indexes’ Variation for the FCM, IT2FCM, and IT2FCM* Algorithms 

One objective of this section is to test the consistency of the improved IT2FCM* with the FCM and IT2FCM methods based on the above-mentioned four validity indexes for different spectral and spatial scales hyperspectral dataset. To realize this, the remote-sensing datasets with different spatial and spectral resolutions were generated from the hyperspectral HYDICE dataset, which has been used to test different classification methods in previous studies [67,73,74,75]. The HYDICE dataset was resampled into 191-band, 97-band, 49-band, 25-band, and 13-band by selecting bands using the Environment for Visualizing Images (ENVI) and Interactive Data Language (IDL, Exelis Inc., Boulder, CO, USA) software programs. The principle of selecting bands is that one of two adjacent bands is removed until the number of all bands remaining is half of the previous number. To conveniently compute the bands, we remove the even-numbered band each time, thus, this produced the 97-band, 49-band, 25-band, 13-band, and until the 7-band images. The spatial resolution of these resized images remained the same as that of the original HYDICE dataset (3 m/pixel). Moreover, datasets with different spatial resolutions were generated by resampling the original HYDICE dataset, which has a spatial resolution of 3 m, to 5 m, 10 m, 15 m, 20 m, and 30 m by using the ENVI software. The spectral resolution of these images was the same as that of the original HYDICE dataset (191 bands). During this process of resampling of spectral and spatial scales, due to simply selecting these bands just through the ENVI software, thus, the noise information was not considered, and the band width remains the same for each spectral channel as the original image, and the SNR also keeps the same. Regarding the resampling of spatial scales, the cubic convolution resampling technique was used when the high-resolution pixel is aggregated into low-resolution pixel; the cubic convolution resampling technique is a useful tool in the ENVI software. Keeping the same SNR and spectral band width and adding no noise information may be more suitable for realizing the goals of this section. The datasets resized into different spectral and spatial scales in this study are listed in Table 5.

#### 4.1.1. Consistency Test for FCM, IT2FCM, and IT2FCM* Algorithms Based on Different Spectral Resolution Hyperspectral Datasets

This section is to test the performance of the FCM, IT2FCM, and IT2CM* algorithms based on validity indexes calculated from the different hyperspectral spectral channel images, and compare the variation of validity indexes of these three fuzzy methods and test their consistency. Table 6 presents the variation of values of validity indexes from above three fuzzy algorithms. From this table, we can find that these values of IT2FCM* have a good consistency with the other two fuzzy methods, but the value from the IT2FCM* algorithm shows a better tendency than the other two methods. This demonstrates that the improved IT2FCM* algorithm behaves with better performance in image clustering than the other two fuzzy methods. In specific, the PC values that were calculated with the improved IT2FCM* algorithm were greater than those of the other algorithms, followed by the values from the FCM algorithm, and the smallest values were those from IT2FCM. The PE values from IT2FCM and the improved IT2FCM* algorithm displayed almost the same trends, and their values were almost equal for the same spectral scales. Compared to the FS values of the other two methods, the FS values for IT2FCM* displayed the most obvious improvement, and IT2FCM* exhibited the smallest values of this metric of all the algorithms for remotely sensed datasets with the same spectral scales. The FS values greatly varied as the spectral scales changed. The XB values from IT2FCM were the smallest for the five datasets with different spectral scales, and the values from the improved IT2FCM* algorithm were smaller than those from FCM. The XB values slightly varied as the spectral scales changes. In general, the validity indexes of PC and FS are significantly improved by the IT2FCM* algorithm, compared to the other two indexes, including PE and XB, thus, the improved IT2FCM* algorithm displayed the best performance, and the IT2FCM algorithm showed better performance than the FCM algorithm. This comparative analysis also showed that the improved IT2FCM* algorithm yielded the best classification results.

#### 4.1.2. Consistency Test for FCM, IT2FCM, and IT2FCM* Algorithms Based on Different Spatial Resolution Hyperspectral Datasets 

In this section, our goal is to comparatively test the performance of the FCM, IT2FCM, and improved IT2FCM* algorithms in different spatial resolution remotely sensed images based on above mentioned validity indexes. Table 7 shows the variation of these values of validity indexes. We can see that, from Table 7, the PC values from the improved IT2FCM* method for remote-sensing datasets with the same spatial scales were the greatest, whereas the FS values were the smallest. The PE values from the IT2FCM and improved IT2FCM* algorithms had slight differences, both of which being smaller than the calculated value from FCM. The trends in XB resembled those of PE, and the XB values from the improved IT2FCM* algorithm were smaller than the values from FCM but slightly larger than the values from IT2FCM. Generally, like the description in above section, the PC and FS indexes are significantly improved using the IT2FCM method, although the other two indexes PE and XB are also slightly improved. However, the values of PE and XB have no large variation, thus, Table 7 shows that the best performance was obtained when using the improved IT2FCM* algorithm, based on this comparative analysis. 

## 5. Conclusions

An improved IT2FCM* algorithm based on type-II fuzzy sets was developed in this paper. This algorithm is intended for use in remote-sensing image classification based on hyperspectral datasets. In the improved type-II fuzzy approach, the ranking of interval number and handling of spectral uncertainty are considered. This is different from those of other fuzzy methods like FCM, IT2FCM, and other traditional supervised classification methods. The advantages of the IT2FCM* algorithm over other methods improve the separability and accuracy of the new method relative to traditional methods. The results also demonstrate this fact. Based on the membership values calculated from the IT2FCM* method, it is notable that this algorithm shows a better separability of different land cover classes. From the results, regarding to the Washington HYDICE, Pavia University, and EO-1 Hyperion hyperspectral image classification, the accuracy of classification of the IT2FCM* algorithm is higher than the accuracy of the FCM and IT2FCM methods. The results also show that the improved IT2FCM* algorithm has optimal performance among these three fuzzy clustering methods due to its separability to produce a finer outputs image of different land-cover types. To comparatively test the performance of FCM, IT2FCM, and improved IT2FCM* algorithms and to test their consistency for different spectral and spatial resolution hyperspectral datasets, four fuzzy validity indexes are introduced. From the results, in general, comparing to the other two fuzzy methods, the value of PC, FS, and XB from the improved IT2FCM* algorithm were improved significantly, and the value of PE had a slight change. This not only demonstrates a good consistency of the IT2FCM* algorithm with FCM and IT2FCM methods, but it also shows that the improved IT2FCM* algorithm behaves with a better performance in image clustering than the other two fuzzy methods. After all, the IT2FCM* is the inheritance and development of IT2FCM. 

Generally, the improved IT2FCM* classification approach showed better separability and accuracy than the traditional FCM and IT2FCM methods. The quantitative performance indexes and graphical outputs demonstrated that the improved IT2FCM* approach significantly outperformed the competing classifiers and is therefore a superior alternative to hyperspectral image classification for use in future research and corresponding applications. However, another problem that needs to be resolved in the future is the computation efficiency. As known to us, regarding to the traditional hard classification methods, nothing about the fuzzy membership needs to be considered, while in the FCM method, the fuzzy membership must be considered during the image clustering, and in the IT2FCM and IT2FCM* algorithms, which are based on type-II fuzzy set, the upper and lower membership degree must be considered. Thus, the computational complexity of IT2FCMs is higher than the normal FCMs. Consequently, the computational efficiencies of the IT2FCM and IT2FCM* algorithms are lower than the computational efficiency of FCM, and are further lower than the computational efficiency of traditional hard classification methods. In addition to the unique advantages of using remote-sensing techniques and hyperspectral remotely sensed datasets for land-cover classification detection, we must be aware of the deficiencies and limitations of this method to better use satellite remote-sensing data. Although satellite-based remote sensing cannot provide information at the high level of detail that is possible in field surveys, this approach provides an alternative for researchers to address the land cover classification to continuously monitor the land-surface change dynamics over a large or local area and provide researchers with valuable, necessary, and complementary information.

In a future study, the IT2FCM* algorithm will be used to more multi/hyperspectral remote-sensing datasets, and its performance with different datasets will be further estimated. This approach will also be used to longtime series of satellite or aerial remotely sensed datasets to further test the separability and to provide an alternative method in addressing the spatiotemporal land cover classification. Besides this, we should be aware that some other materials should be considered to better use multi/hyperspectral remote-sensing techniques to monitoring LULC dynamics based on image classification. Aerial photographs or more field-survey data for additional years should be collected to strengthen and evaluate the results, which is especially important for remote-sensing image clustering, although these data are rarely collected because of economic limitations and a lack of some necessary equipment. Due to the computational complexity of IT2 FS, the computational complexity of IT2 FCMs is higher than normal FCMs. It is very important to improve their computational efficiency, and we will study this problem in the next step. Recently, many researchers have used the nearest-neighbor method combined with spatial information to optimize the IT2FCM algorithm, but the scale effect has never been considered. Therefore, in future work, the scale effect of the surface will be considered, and the spatial information will be utilized to further optimize the IT2FCM* algorithm.

## Figures and Tables

**Table 1:** Detailed process of the IT2FCM* algorithm.

Main	Detail
Step 1. Initialization of the process.	1.1 Selection of the parameters *m*1 and *m*2 (1 < *m*1 < *m*2), the termination criterion value σ and the number of clustering types T.
	1.2 Initialization of the lower membership and upper membership grade matrix $\tilde{u}=\left[\underline{u},\bar{u}\right]$ based on a random method.
Step 2. Computation of all the centroids $\tilde{v}=\left[{\tilde{v}}_{i}\right]$, ${\tilde{v}}_{i}\in R^{I}$, *i* = 1, 2, ..., *C* and updating of their respective lower and upper membership grade matrix.	2.1 Computation of all the centroids $\tilde{v}=\left[{\tilde{v}}_{i}\right]$, ${\tilde{v}}_{i}\in R^{I}$, *i* = 1, 2, ..., *C* and determining their respective lower and upper bands ${\tilde{v}}_{i}^{L}$ and ${\tilde{v}}_{i}^{R}$ by using the KM method.
	2.2 Calculation of the Euclidean distance between interval vectors using Equation (9).
	2.3 Updating of the respective lower membership and upper membership grade matrix $\tilde{u}=\left[\underline{u},\bar{u}\right]$ using Equation (10).
	2.4 Calculate the objective function using the Equation (11), if Equation (12) is satisfied, then go to next step, otherwise, go on iteration based on this step 2.
Step 3. Classification of each sample using interval-number-ranking method and by considering the optimal fuzzy membership value.	3.1 Calculation of possibility matrix based on Equation (14).
	3.2 Calculation of the ranking vector $w_{k}={\left(w_{1k},w_{2k},\ldots ,w_{Tk}\right)}^{Transpose}$ based on the possibilty matrix in step 3.1. (more details refer to [61])
	3.3 Assigning a sample to a cluster according to the index of maximum value in the ranking vector in step 3.2.
	3.4 Outputs of the clustering results in step 3.3 based on optimal fuzzy membership value.

**Table 2:** The accuracy of the classification results of HYDICE dataset based on FCM, IT2FCM, and IT2FCM* algorithm.

Class	FCM		IT2FCM		IT2FCM*	
	Prod. Acc.	User Acc.	Prod. Acc.	User Acc.	Prod. Acc.	User Acc.
Water	100	97.67	100	98.11	100	99.94
Sparse grassland	96.00	75.77	97.85	83.50	98.86	94.86
Dense grassland	91.51	81.05	91.36	92.73	95.74	97.85
Trees	91.40	94.90	94.01	96.13	95.48	99.91
Roads	92.97	75.65	92.98	81.50	97.81	93.12
Buildings and bare soil	82.26	96.76	88.10	98.13	93.82	97.43
Shadow	95.55	95.27	96.38	96.18	96.17	98.98
Overall accuracy	86.70		90.57		96.23	
Kappa coefficient	0.84		0.88		0.95	

Prod. Acc. = Product accuracy, User acc. = User accuracy.

**Table 3:** The accuracy of the classification results of Pavia University based on the FCM, IT2FCM, and IT2FCM* algorithms.

Class	FCM		IT2FCM		IT2FCM*	
	Prod. Acc.	User Acc.	Prod. Acc.	User Acc.	Prod. Acc.	User Acc.
Asphalt	83.46	78.30	89.32	78.55	96.13	98.93
Meadows	65.31	60.54	80.56	90.12	97.01	88.56
Trees	78.26	81.67	90.24	97.26	95.35	97.30
Painted metal sheets	85.43	86.75	94.33	83.65	98.87	95.26
Bare soil	64.83	57.03	89.45	80.92	95.57	97.05
Shadows	48.28	51.36	91.37	93.55	96.10	94.27
Overall Accuracy	69.52		89.25		96.52	
Kappa Coefficient	0.61		0.85		0.94	

**Table 4:** The accuracy of the classification results of Hyperion dataset based on FCM, IT2FCM, and IT2FCM* algorithm.

Class	FCM		IT2FCM		IT2FCM*	
	Prod. Acc.	User Acc.	Prod. Acc.	User Acc.	Prod. Acc.	User Acc.
Water	87.61	99.92	90.52	94.52	95.60	99.82
Impervious surface	85.48	99.88	87.38	93.28	91.09	99.88
Bare soil	92.45	76.31	93.28	92.98	97.45	78.85
Grassland	91.59	92.49	91.47	91.56	93.59	91.49
Cropland	95.94	94.63	95.94	87.23	96.86	94.62
Overall Accuracy	89.09		93.26		95.82	
Kappa Coefficient	0.82		0.87		0.94	

**Table 5:** Remote-sensing datasets with different spectral and spatial scales.

Data Type	Spectral Bands	Spatial Resolution (m)
Datasets with different spectral resolutions	191	3
	97	3
	49	3
	25	3
	13	3
	7	3
Datasets with different spatial resolutions	191	3
	191	5
	191	10
	191	15
	191	20
	191	30

**Table 6:** Type-I fuzzy cluster validity indexes for the IT2FCM*, IT2FCM, and FCM algorithms that were applied to remotely sensed datasets with different spectral scales.

Number of Spectral Channels	Index	FCM	IT2FCM	IT2FCM*
191 bands	PC	0.206	0.178	0.235
	PE	1.846	1.756	1.746
	FS	−5.698 × 10^8^	−3.045 × 10^8^	−6.191 × 10^8^
	XB	0.284	0.182	0.210
97 bands	PC	0.217	0.208	0.216
	PE	1.845	1.760	1.766
	FS	−4.078 × 10^8^	−2.858 × 10^8^	−4.576 × 10^8^
	XB	0.284	0.196	0.208
49 bands	PC	0.207	0.178	0.236
	PE	1.844	1.757	1.761
	FS	−2.868 × 10^8^	−1.989 × 10^8^	−3.196 × 10^8^
	XB	0.291	0.199	0.232
25 bands	PC	0.207	0.178	0.236
	PE	1.845	1.757	1.761
	FS	−1.908 × 10^8^	−1.334 × 10^8^	−2.151 × 10^8^
	XB	0.301	0.208	0.241
13 bands	PC	0.210	0.179	0.239
	PE	1.837	1.753	1.754
	FS	−1.619 × 10^8^	−1.104 × 10^8^	−1.784 × 10^8^
	XB	0.287	0.190	0.221
7 bands	PC	0.205	0.180	0.233
	PE	1.844	1.768	1.765
	FS	−1.095 × 10^8^	−0.745 × 10^8^	−1.181 × 10^8^
	XB	0.571	0.201	0.205

**Table 7:** Type-I fuzzy cluster validity indexes when applying the IT2FCM*, IT2FCM, and FCM algorithms to remotely sensed datasets with different spatial scales.

Spatial Resolution	Index	FCM	IT2FCM	IT2FCM*
5 m	PC	0.203	0.177	0.234
	PE	1.854	1.761	1.766
	FS	−1.968 × 10^8^	−1.416 × 10^8^	−2.270 × 10^8^
	XB	0.282	0.196	0.208
10 m	PC	0.205	0.177	0.236
	PE	1.851	1.758	1.763
	FS	−5.038 × 10^7^	−3.611 × 10^7^	−5.790 × 10^7^
	XB	0.289	0.197	0.209
15 m	PC	0.203	0.177	0.235
	PE	1.855	1.759	1.764
	FS	−2.150 × 10^7^	−1.574 × 10^7^	−2.559 × 10^7^
	XB	0.274	0.215	0.227
20 m	PC	0.197	0.175	0.231
	PE	1.870	1.768	1.776
	FS	−1.151 × 10^7^	−0.861 × 10^7^	−1.391 × 10^7^
	XB	0.283	0.200	0.232
30 m	PC	0.201	0.177	0.235
	PE	1.860	1.767	1.766
	FS	−5.361 × 10^6^	−3.805 × 10^6^	−6.491 × 10^6^
	XB	0.288	0.194	0.207
